# Polymer-fiber-coupled field-effect sensors for label-free deep brain recordings

**DOI:** 10.1371/journal.pone.0228076

**Published:** 2020-01-24

**Authors:** Yuanyuan Guo, Carl F. Werner, Andres Canales, Li Yu, Xiaoting Jia, Polina Anikeeva, Tatsuo Yoshinobu

**Affiliations:** 1 Frontier Research Institute for Interdisciplinary Sciences (FRIS), Graduate School of Medicine, Graduate School of Biomedical Engineering, Tohoku University, Sendai, Miyagi 9800845, Japan; 2 Department of Electronic Engineering, Department of Biomedical Engineering, Tohoku University, Sendai, Miyagi, 9808579, Japan; 3 Department of Materials Science and Engineering, Research Laboratory of Electronics, and McGovern Institute for Brain Research, Massachusetts Institute of Technology, Cambridge, MA 24139, United States of America; 4 Bradley Department of Electrical and Computer Engineering, Virginia Polytechnic Institute and State University, Blacksburg, VA 24060, United States of America; Nicolaus Copernicus University, POLAND

## Abstract

Electrical recording permits direct readout of neural activity but offers limited ability to correlate it to the network topography. On the other hand, optical imaging reveals the architecture of neural circuits, but relies on bulky optics and fluorescent reporters whose signals are attenuated by the brain tissue. Here we introduce implantable devices to record brain activities based on the field effect, which can be further extended with capability of label-free electrophysiological mapping. Such devices reply on light-addressable potentiometric sensors (LAPS) coupled to polymer fibers with integrated electrodes and optical waveguide bundles. The LAPS utilizes the field effect to convert electrophysiological activity into regional carrier redistribution, and the neural activity is read out in a spatially resolved manner as a photocurrent induced by a modulated light beam. Spatially resolved photocurrent recordings were achieved by illuminating different pixels within the fiber bundles. These devices were applied to record local field potentials in the mouse hippocampus. In conjunction with the raster-scanning via the single modulated beam, this technology may enable fast label-free imaging of neural activity in deep brain regions.

## Introduction

Systems neuroscience aims to correlate observed behaviors to underlying cellular and circuit dynamics. Electrophysiological recordings with miniaturized electrodes arrays capture neural dynamics at the single-neuron and population levels and correlate it to behavior [1–3]. By leveraging advances in complementary metal-oxide-semiconductor (CMOS) processing, neural probes can simultaneously record activity of hundreds of neurons [1, 3]. However, these probes do not reveal structural connectivity within the circuit. In contrast, optical imaging allows for direct visualization of large neuronal ensembles [4], and with the introduction of miniaturized endoscopes, its use can be extended from superficial cortical regions to deep brain structures [5–8]. Optical imaging relies on fluorescent activity indicators, which are introduced via genetic means with cell-type specificity [9] or infused exogenously [10] which indiscriminately labels all cell types. While genetically encoded activity indicators are used routinely in rodents, their delivery and robust expression remain challenging in other organisms. Furthermore, the spectral properties and kinetics of both the genetically encoded and synthetic activity indicators limit the spatiotemporal resolution of optical imaging. Even with the multiphoton excitation techniques the imaging depths are restricted to < 2 mm, close to the bulky collection optics [5, 8]. Consequently, there remains a need for tools capable of temporally precise electrophysiological recordings of spatially identifiable neural networks in deep brain regions.

Here, we introduce a miniature hybrid device based on field-effect sensors and flexible polymer fibers to realize multi-spot recordings for obtaining brain electrophygiological activities. The field-effect has previously enabled measurements of electrophysiological potentials in neuronal cultures and brain tissue slices [3, 11–13]. In these experiments, the trans-membrane current from a neuron in the electrolyte polarizes the gate dielectric of an ion-sensitive field-effect transistor (ISFET), which results in a change in the conductance of the underlying space charge region within the silicon channel. The applications of these pioneering tools in deep brain structures *in vivo*, however, remain limited by the need for these probes approaching to the neurons of interest.

**Fig 1:**
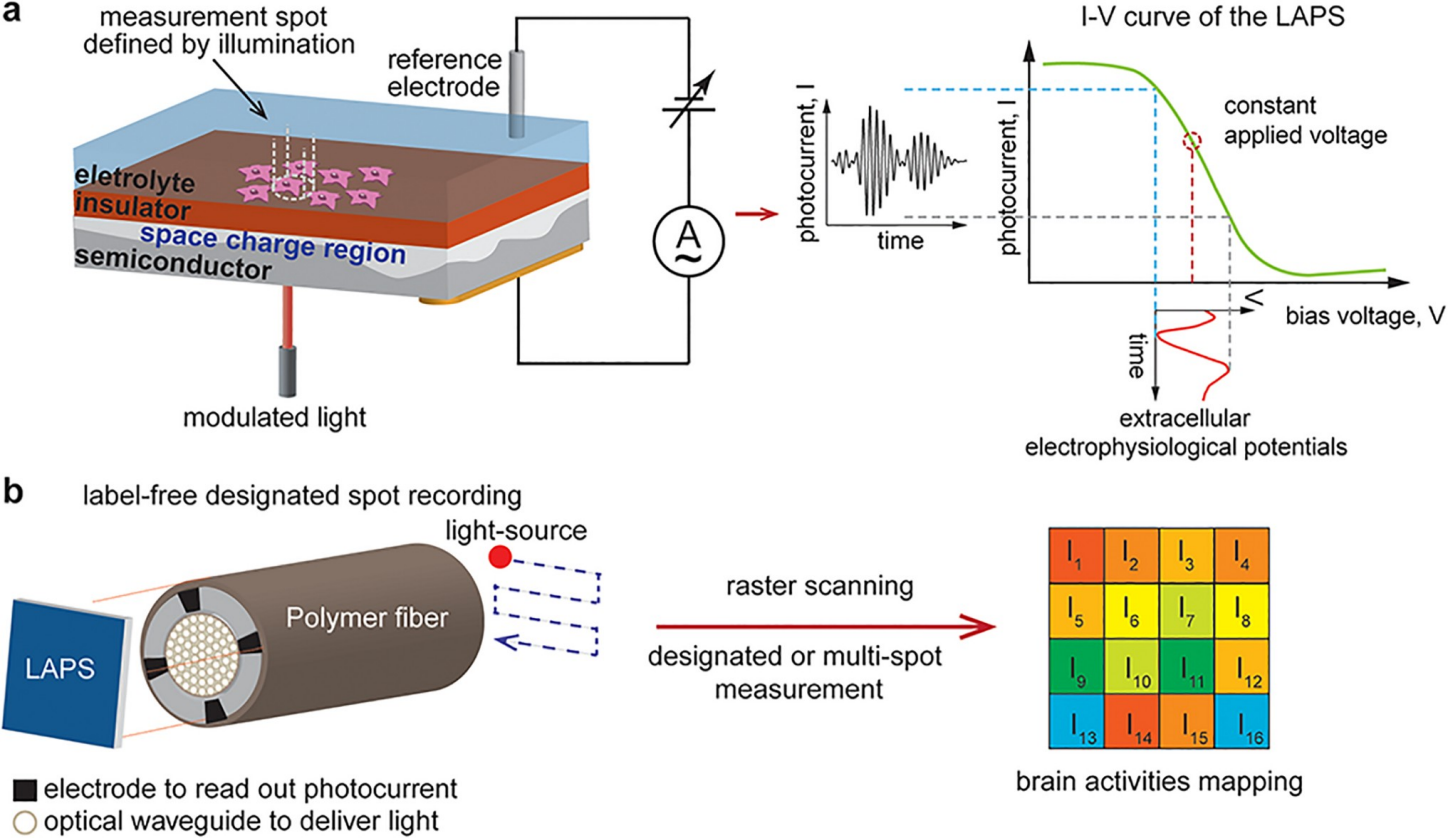
Schematics of the light-addressable potentiometric sensor (LAPS) and the proposed miniature hybrid device. **a.** An illustration of the LAPS and its light-addressing working principle show the photocurrent can ‘sample’ the extracellular electrophysiological potentials. **b.** The mapping of neuronal dynamics via the proposed label-free hybrid device is realized via raster-scanning of the light source. Thus, the information of neuronal dynamics at different measurement spots can be deciphered by the localized photocurrent induced by the focused light beam.

To address this challenge and to enable spatially resolved electrophysiological recording in deep brain structures, we developed a platform that leverages the characteristics of light-addressable potentiometric sensors (LAPS) [14–17]. In these field-effect devices, the surface potential at the electrolyte-insulator interface in response to the brain electrical potential is measured in the form of a photocurrent induced by a modulated light beam, which illuminates the backside of the electrolyte-insulator-semiconductor (EIS) sensor (Fig 1a). Electron-hole pairs photogenerated in the bulk semiconductor diffuse towards the space charge region, where they are separated by the electric field, thereby producing a transient current. When the light is turned off, a reverse transient current flows. Therefore, pulsed illumination produces an alternating photocurrent, the amplitude and phase shift of which depend on the capacitance of the local space charge region and, in turn, on the local surface potential. In this architecture, illumination spot size on the sensing surface determines the resolution of the local field measurement. By scanning LAPS surface via a focused light beam, the spatial distribution of the electrophysiological potentials in the brain can be obtained. An alternative way can be illuminating multiple locations on the LAPS surface with light beams modulated at different frequencies, the resulting photocurrents can be demultiplexed to obtain electrophysiological mapping. However, due to the restriction from the planar structure of the LAPS, current measurement systems of the LAPS, based on either raster-scan of a focused laser beam [18–20] or the multi-fiber coupled multiplexed illumination [21], are considerably bulky and mainly used in *in vitro* biological applications.

To facilitate the application of LAPS *in vivo*, these devices must be coupled to the light delivery and photocurrent acquisition capabilities. To enable delivery of either a single focused light beam or array of modulated light beams onto LAPS surfaces and to establish an electrical interface with the external world, we leveraged a versatile thermal drawing process [22–24] to fabricate flexible fibers consisting of an all-polymer optical waveguide bundle core surrounded by an array of low-melting temperature bismuth-tin (BiSn) alloy electrodes within a polymer cladding. The resulting devices were fabricated by connecting LAPS chips to the optoelectronic fibers (Fig 1b). These fiber-coupled LAPS permitted multi-spot photocurrent recordings, and further the label-free electrophysiological recording of neural activity both in superficial and deep brain regions. Polymer fibers with similar mechanical properties have been previously shown to produce minimal foreign body response [23, 24], which indicates that the described device may enable electrophysiological recording in a spatially resolved manner of deep brain structures over extended periods of time.

## Materials and methods

### Miniaturized LAPS chip preparation

For this study, the miniaturized LAPS chip preparation required multiple steps. We first obtained two types of standard silicon wafers (6″) doped with boron with thickness of both 200 μm and 100 μm, each covered with 50 nm of silicon dioxide and 50 nm of silicon nitride (KST World Corp.). Then, the thicker wafer was grinded with diamond paste from the backside to achieve thinned silicon layer less than 100 μm. Thinning the silicon layer served three purposes, namely the improvement of the spatial resolution of the LAPS by limiting the lateral diffusion of the photocarriers, the increase of the detected photocurrent by shortening the travel distance of photocarriers and decreasing their loss from recombination [25, 26], and finally to offer a rough surface which would facilitate easy adhesion of metal contact. Afterwards, layers of titanium and gold with thickness of a few nanometers each were thermally evaporated to the backside to form an ohmic contact. In the meanwhile, the optical transparency for the illumination was still maintained.

**Fig 2:**
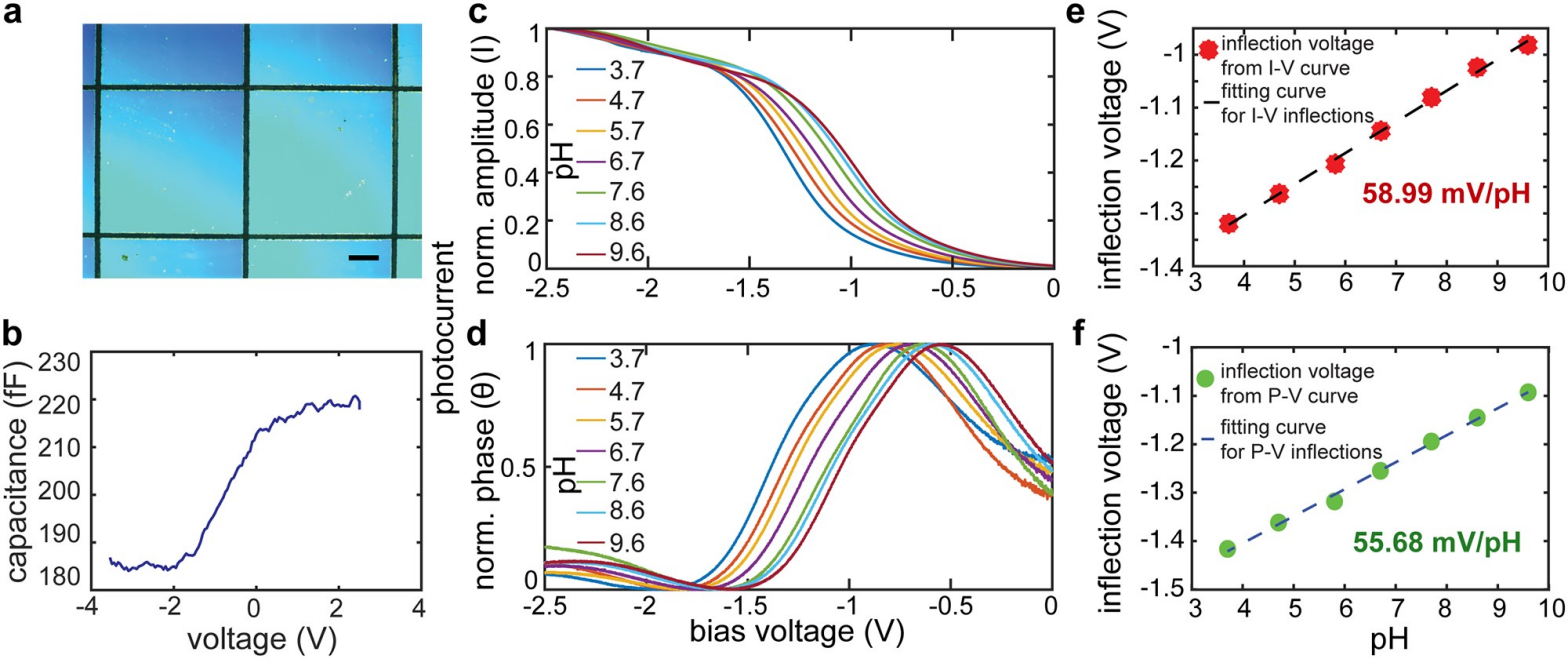
Characterization of the LAPS chips. **a.** Optical microscope image of the diced LAPS chips (scale bar = 200 μm) **b.** Capacitance versus voltage curve of the miniaturized LAPS chip indicating its inversion, depletion and accumulation region. **c-d.** Normalized amplitude (I, **c**) and phase (*θ*, **d**) of photocurrent versus bias voltage (V) in response to solutions with different pH. **e-f.** The pH sensitivity characterized from the I-V (**e**) and *θ*-V (**f**) curves.

Lastly, the resulting wafer was mounted on a UV tape (UHP-0805M6, DENKA company) and diced using a diamond blade (Z09-SD4000-Y1-90) and dicing saw (DISCO DAD 322). The spindle speed was fixed at 30000 rpm and the feeding speed was in the range of 5 to 10 mm/s. The resulting miniaturized LAPS chips had dimensions of 1 mm × 1 mm, 800 μm × 800 μm, or 500 μm × 500 μm (Fig 2a). In addition to dicing, we also cut the manual cutting of the silicon chips into desired sizes down to 1 mm.

### Evaluation of the pH sensitivity of LAPS chip

The backside of the LAPS chip was illuminated by focusing an infrared laser (800 nm) modulated at 1 kHz. By varying the LAPS bias voltage from -2.5 to 0 V with an increment step of 2 mV, photocurrent versus bias voltage responses to solutions with pH of 3.7, 4.7, 5.8, 6.7, 7.7, 8.6 and 9.6 were obtained. Then, the pH sensitivity was calculated from the inflection voltage for each pH buffer (Fig 2c–2f).

### Multifunctional fiber fabrication via thermal drawing process

**Fig 3:**
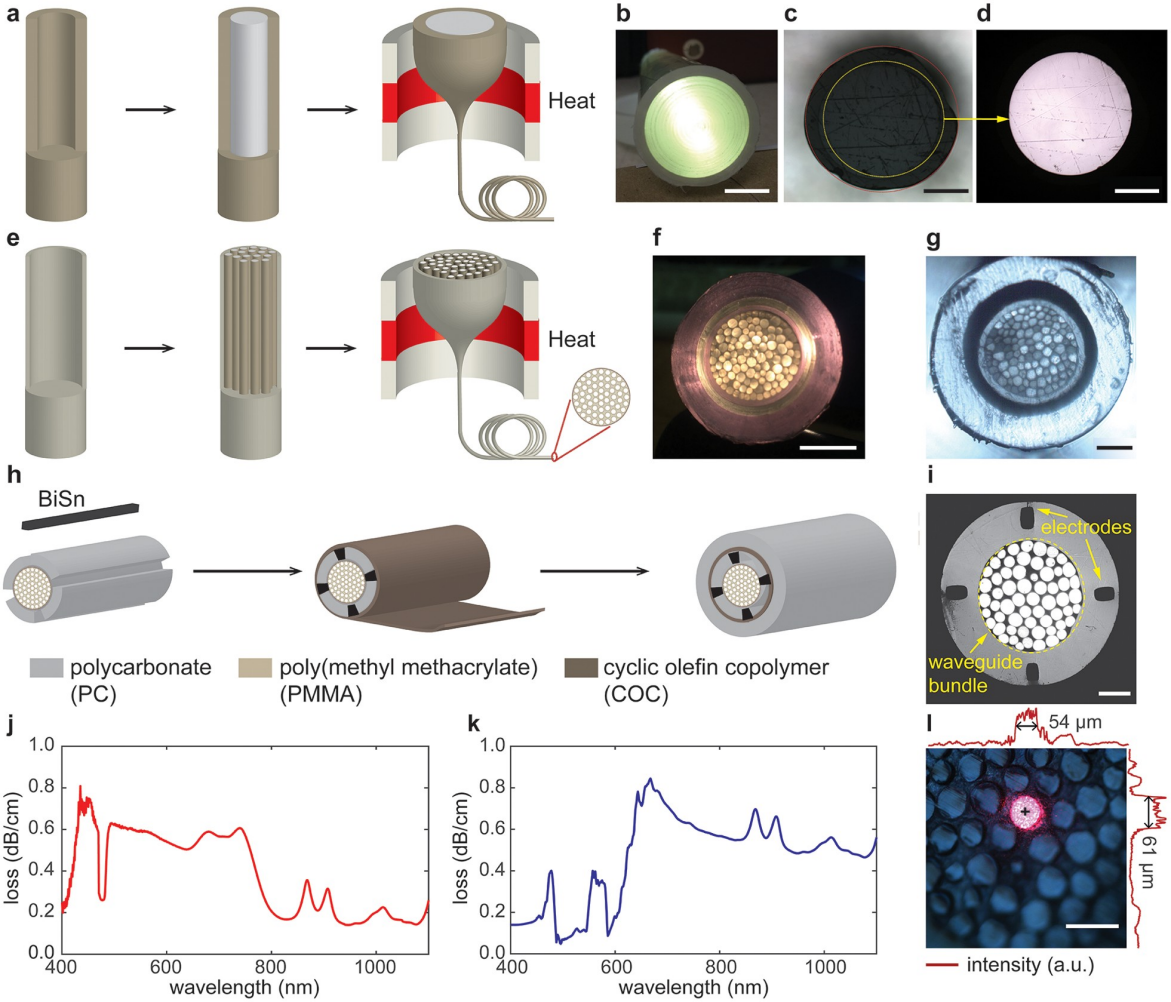
Fiber fabrication and characterization. **a.** An illustration of the first step of the thermal drawing process. **b.** A photograph of the PC/PMMA preform. **c-d.** Optical microscope images of the thermally drawn PC/PMMA fiber without (**c**) and with (**d**) light confined within the PC core. Scale bar = 200 μm. **e.** An illustration of the second step of the thermal drawing process. **f-g.** A photograph of the preform (scale bar = 1 cm, **f**) and an optical microscope image of the thermally drawn fiber (**g**) with 108-waveguide bundle. Scale bar = 100 μm. **h.** An illustration of the fabrication process for the multifunctional fiber with an optical-waveguide bundle core and bismuth-tin alloy (BiSn) electrodes. **i.** An optical microscope image of the thermally drawn fiber illustrated in (**h**). Scale bar = 100 μm. **j.** An optical loss spectrum of a single PC/PMMA optical fiber. **k.** An optical loss spectrum of the fiber with a bundle of 108 PC/PMMA waveguides. **l.** An optical micrograph of light confinement within a single waveguide. Light intensity distribution profiles are shown in upper and left panels. Black cross indicates the illuminated waveguide center. Scale bar = 100 μm.

Multi-step drawing was adopted for the multifunctional fibers. For the optical waveguide, polycarbonate (PC) and poly(methyl methacrylate) (PMMA) were chosen as a core material and a cladding material, respectively. They have similar processing temperature (T_g,PC_ = 145 °C, T_g,PMMA_ = 105 °C) but significant difference in the refractive index (n_PC_ = 1.58 and n_PMMA_ = 1.48). The preform consisted of a PC rod with diameter of 25.4 mm (McMaster-Carr Supply Company) and a PMMA tube (U.S. Plastic Corp.) with outer diameter of 28.575 mm and inner diameter of 25.4 mm, which were consolidated at 150 °C in vacuum for 20 minutes. The preform was then drawn, (Fig 3a–3d and S1 Fig), which produced a polymer optical fiber.

Afterwards, 108 sections of this fiber with diameter of approximately 1 mm and length of 25 cm were cut and bundled together with a layer of PC rolled tightly around them. In order to maintain a stable and controlled drawing process, a layer of cyclic olefin copolymer (COC) (TOPAS Advanced Polymers GmbH) as an etching stop layer followed by another layer of PC as a sacrificial layer was rolled to increase the diameter of the resulting preform to 28 mm. This preform was consolidated at 180 °C in vacuum for 20 minutes and went through another round of thermal drawing, resulting in a fiber which can be used for optical imaging (Fig 3e–3g).

To fabricate a fiber with a waveguide bundle and electrodes, a layer of PC was rolled around a bundle of 60 PC/PMMA optical fibers and consolidated at 180°C in vacuum. We then machined four slots with dimensions of 2 mm × 1 mm × 8 cm on the PC of the preform, where 58bismuth-42tin ribbons (The Indium corporation) were inserted. A thin layer of PC film was rolled on top, followed by a COC layer and another PC layer to achieve a preform size of 28 mm. It then went through a second thermal drawing (S2 Fig), resulting in a fiber with 60 pixels and 4 BiSn electrodes (Fig 3h–3i).

### Optical transmission measurement

The PC/PMMA optical fiber with a diameter close to 670 μm and the fiber with 108-PC/PMMA-waveguide bundle with 670 μm diameter were connected to optical ferrules (Thorlabs) and polished with a SpecPro Polisher (Krell Technologies). Their spectral attenuation was measured using a cutback method and an optical spectrum analyzer (AQ-6315A). The cutback method is to compare the optical power, P_1_, transmitted through a longer length of fiber, L_1_, to the power, P_2_, transmitted through a shorter length of the fiber, L_2_, without disturbing the input conditions. The attenuation, *α*, of the tested fiber (dB/m) is calculated based on the following equations.

$$\alpha =\frac{10}{L_{1}-L_{2}}\times \log_{10}\left(\frac{P_{1}}{P_{2}}\right)$$

### Characterization of the micro-LAPS chip

A probing station (Keithley 4200SCS Parameter Analyzer), equipped with two micromanipulators with two needle probes was used to contact the frontside and the backside of LAPS. A DC voltage sweep from -3.5 V to 2 V was applied from the frontside of the LAPS to the backside, and a 10 mV sinusoidal voltage was superimposed to the DC voltage. The resulting AC current was recorded, from which the capacitance of the LAPS chip was calculated.

### Fabrication of the multifunctional fiber-coupled LAPS device

**Fig 4:**
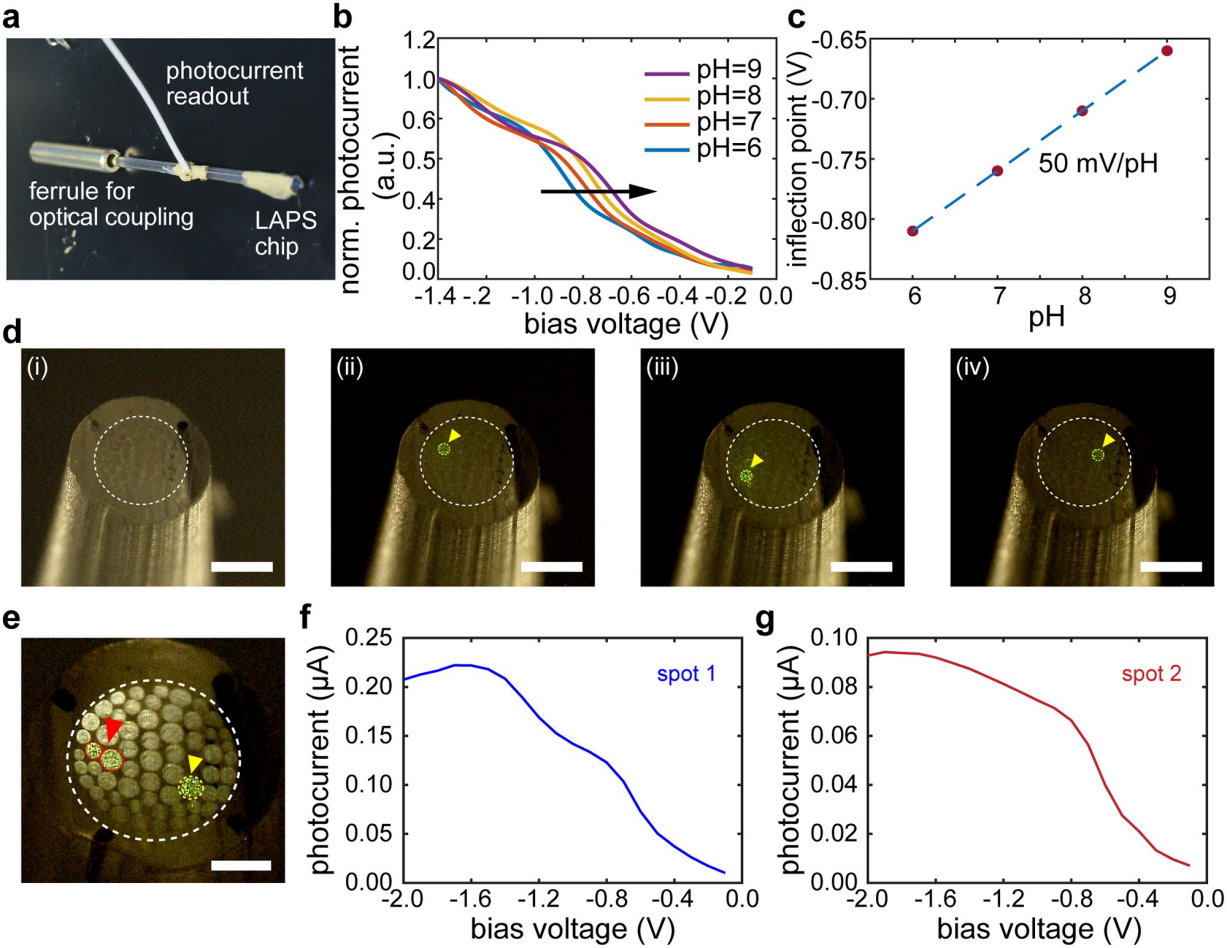
Characterization of the fiber-coupled LAPS device. **a.** A photograph of a fiber-coupled LAPS endoscope and its connections. **b.** Normalized photocurrent amplitude (I) vs. voltage (V) recorded by the endoscope in solutions with pH 6, 7, 8, and 9 as indicated by the black arrow. **c.** The pH sensitivity of the endoscope calculated from the inflection point of the I-V curves in (**b**). **d.** Optical micrographs of a multifunctional fiber (i) with light addressing different single pixels indicated by the yellow arrows (ii-iv). Scale bar = 500 μm. **e.** An optical micrograph of a multifunctional fiber with light addressing two different locations as indicated by yellow and red arrows. Scale bar = 250 μm. **f-g.** Measurements of localized photocurrent responses of the device with light illuminating different spots. Scale bar = 100 μm.

The LAPS chip was connected to the tip of the multifunctional fiber with silver paint (SPI supplies, Inc.). The exposed edges of the LAPS chip were then covered with medical epoxy (McMaster-Carr). At the backend of the fiber, the electrode was exposed from the fiber side by removing the polymer cladding with a surgical scalpel under a microscope and connected to a copper wire using silver paint. The free end of the copper wire was soldered to a pin connector. An optical ferrule 6.5-mm-long, with a 2.5-mm-outer-diameter and 1-mm-inner-diameter was connected to the fiber with optical epoxy (Thorlabs) and cured overnight. The ferrule end was polished with a Thorlabs fiber polishing kit to achieve optimal optical coupling (Fig 4a).

### Multifunctional fiber-coupled LAPS microscopy device characterization

The pH sensitivity of the device was characterized by addressing a single pixel with an infrared diode laser (800 nm) modulated at 1 kHz. A bias voltage was applied from -1.4 to -0.1 V with an increment step of 0.1 V, and the photocurrent versus bias voltage responses were obtained for solutions with pH of 6, 7, 8, and 9, from which the pH sensitivity was calculated (Fig 4b and 4c).

Multi-spot addressability by light was achieved by using the preform-to-fiber region from the thermal drawing process. Thus, multiple lights can be delivered into small areas with distinct spatial resolution (Fig 4d–4g).

### Fabrication of the LAPS coupled to fiber with a ring of electrodes

In addition to the newly developed multifunctional fiber, a fiber with a ring of tin electrodes and a hollow core (S3 Fig) was also used to combine with the LAPS. Similarly, the LAPS chip was connected to the tip of the fiber via silver paint (SPI supplies, Inc.). Then, an optical fiber was inserted into the central hollow core for light delivery to the LAPS while the electrodes led out the photocurrent. This device was further evaluated for proof-of-principle electrophysiological recordings.

### Mouse surgery and electrophysiological recording

All animal procedures were approved by the Committee on Animal Care and Use of Massachusetts Institute of Technology and carried out in accordance with the National Institutes of Health Guide for the Care and Use of Laboratory Animals. Male C57BL/6 mice aged 7 to 9 weeks (Jackson Laboratory) were used for this experiment, and all surgeries were conducted under aseptic conditions. The recordings were obtained when the mice were under deep anesthesia at the time of surgical implantation (in mg/kg bodyweight: ketamine, 100; xylazine, 10; in saline) and positioned in a stereotactic frame (David Kopf Instruments). The scalp was exposed via a skin incision, where lambda and bregma points were used to align the skull with respect to a mouse brain atlas. The device was first put on the surface of the brain after drilling a hole through the skull, and a stainless-steel wire was put on the neck muscle, serving as reference and ground. The device was then implanted in the hippocampal formation (HPF, coordinates relative to bregma; -2 mm anteroposterior (AP); -1.5 mm mediolateral (ML); -1.9 mm dorsoventral (DV)). In case for the acute surgeries, the devices can be cleaned afterwards with ethanol and go through gas sterilization with ethylene oxide. They can be readily re-used for the next experimental sessions. In addition, the cost of a single device is neglectable with an estimate of less than $5, thus they can be disposed in case for the chronic implantation.

**Fig 5:**
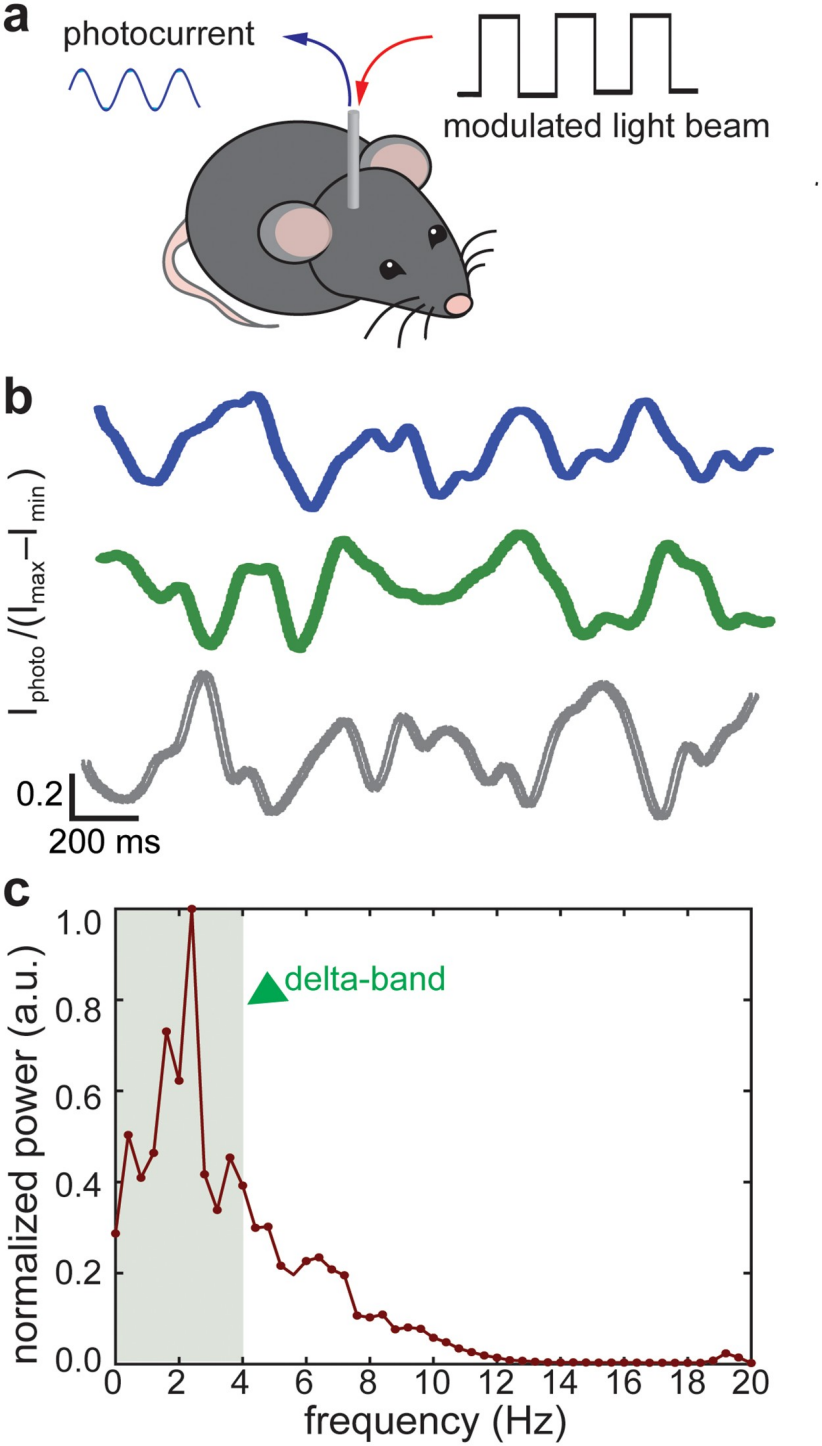
Evaluation of the fiber-coupled LAPS device *in vivo* for electrophysiological recordings. **a.** The schematic illustrating experiments *in vivo*. **b.** Representative traces of photocurrent amplitude recorded in the mouse hippocampus. **c.** Power spectrum of the local field potentials recorded in the hippocampus of an anesthetized mouse. The green arrow points to the delta frequency band.

A 473 nm diode-pumped solid-state laser (OEM Laser Systems) coupled to the optical fiber was used as the light source for the LAPS chip. The light was modulated at 200 Hz; thus, the resulting photocurrent had a base frequency of 200 Hz, the amplitude of which was in response to brain activities on the sensor surface (Fig 5).

We calculated the signal amplitude with a lock-in amplifier algorithm with a 200 Hz fixed reference frequency. The amplified signal then went through a low pass FIR filter with cut-off frequency of 20 Hz. The power spectral analysis was determined with a Fast Fourier Transform (FFT) algorithm.

## Results

### Characterization of LAPS

Miniaturized LAPS chips were prepared from commercially available standard n-type silicon wafers with silicon oxide and silicon nitride sensing layers. Ohmic contacts to the LAPS sensors were established by evaporating thin layers of titanium and gold onto the backside of the wafers (Fig 2a). The chips were then examined for their field-effect behavior, and as expected, LAPS chips exhibited inversion, depletion, and accumulation regimes shown in the capacitance-voltage characteristic (Fig 2b). To assess the sensitivity of LAPS to local charge, we examined their photocurrent behavior in response to pH changes (Fig 2c). Based on the inflection voltage, which is calculated from either amplitude or phase characteristics of the photocurrent in response to different pH, sensitivities of 58.99 mV/pH and 55.68 mV/pH respectively were measured, which are close to the Nernstian value (59.17 mV/pH at 25°C, Fig 2d).

### Characterization of multicore polymer fibers

A multifunctional fiber with a waveguide bundle core was produced via a two-step thermal drawing process (Fig 3). First, a polymer optical fiber consisting of polycarbonate (PC) core and poly(methyl methacrylate) (PMMA) cladding was drawn from a macroscale preform (Fig 3a). By changing mainly the tension on the fiber along with the temperature during the drawing process, the core and cladding dimensions were tuned between 650-1690 μm and 55-150 μm, respectively (S1 Fig). PC and PMMA were chosen for their similar glass transition temperatures (T_g,PC_ = 145 °C, T_g,PMMA_ = 105 °C), which permits their co-drawing, and significantly different refractive indices (n_PC_ = 1.58 and n_PMMA_ = 1.48) necessary for low-loss optical guidance (Fig 3b–3d).

During the second step of the fabrication process, we prepared a preform containing 108 sections of the PC/PMMA optical fibers with diameters between 1-2 mm drawn in the first step(Fig 3e). This preform was then drawn into kilometers of fiber comprised of 108 independent waveguides with diameters 8 μm to 20 μC, which represents a size reduction in excess of 3000 from the dimensions of the first-step preform(Fig 3f and 3g). This fiber could be used to deliver a plurality of multiple modulated light beams to LAPS.

In addition to the multicore waveguide, the LAPS-based microscope had to be outfitted with electrodes for the photocurrent readout. Consequently, we engineered another preform containing 60 PC/PMMA optical fibers drawn in the first step and 4 bismuth-tin (BiSn) ribbons as electrodes (Fig 3h). After the second thermal drawing step, the fiber included 60 optical waveguide pixels with diameters of 20 μm to 60 μC to deliver light beams to LAPS and four BiSn electrodes with dimensions 30±3 × 45±5 μm^2^ for the photocurrent readout (Fig 3i).

Optical properties of PC/PMMA fibers produced in the first step and those of the multicore fibers were characterized to corroborate their utility to deliver light powers sufficient to drive LAPS. PC/PMMA optical fibers with outer diameters of 670 μm (567 μm core) exhibited relatively smooth absorption spectra in the wavelength range between 500 nm and 1100 nm (Fig 3j). These devices exhibited power loss <0.8 dB/cm, which is comparable to previously reported fibers with PC cores [24] and is suitable for light transmission at centimeter scales.

As expected, second-step PC/PMMA fibers with 108 cores exhibited flat absorption spectra similar to those of the single-strand fiber precursors ((Fig 3k), and optical losses of <0.85 dB/cm. As the light confinement within a single waveguide core in a multicore fiber determines the imaging resolution of the fiber-coupled LAPS microscopes, we further examined the light transmission by individual pixels. To that end, we found that the light coupled into a single pixel of a multicore fiber propagates predominantly within the chosen core without evanescent cross-talk with the neighboring cores (Fig 3l). This confinement was demonstrated by the optical intensity profiles. In addition to optical properties of the optical cores, we have measured the resistance of the BiSn electrodes, which was 5±1.5 Ω for 1 cm long fibers consistent with the bulk electrical resistivity of this alloy (30 μΩ cm to 35 μΩ cm).

### Characterization of fiber-coupled LAPS devices

Each LAPS chip (Fig 2a) was integrated at the tips of a multifunctional fiber (Fig 3i), and the backend of the latter was connectorized to a ferrule for optical coupling and to an insulated microwire for the photocurrent readout (Fig 4a).

We exploited the proton-sensing feature of LAPS to corroborate the functional performance of these devices following their integration with the multifunctional fibers. We found pH sensitivity of 50 mV/ pH for the fiber-coupled LAPS (Fig 4b and 4c), a value consistent with standalone devices (Fig 2c), which is also close to the Nernstian limit.

We next assessed the multi-spot addressability afforded by the waveguide bundle within the core of the multifunctional fibers ((Fig 4d). Micrographs of the polished fiber cross sections demonstrate independent optical coupling into different waveguides within the bundle. Notably, multiple pixels can be addressed simultaneously and independently as shown in an example of two distinct pixels (Fig 4e). Furthermore, the photocurrent responses (Fig 4f and 4g) localized to the illuminated spots on the LAPS sensing surface highlight the multi-spot measurements. And with further calibration in standard buffer solutions, potential utility of this imaging/mapping platform in accurately measuring localized biochemical signal can become possible.

### *In vivo* electrophysiological recording with fiber-coupled LAPS Ddevices

To demonstrate the usefullness of the fiber-coupled LAPS for label-free electrophysiological recording and further potential imaging, we conducted a proof-of-concept evaluation of these devices in mice. The devices with LAPS chips at the tips of fibers with a ring of electrodes for photocurrent readout and a hollow channel, which could fit an optical waveguide, were implanted into the hippocampus of anesthetized wild type (WT) mice (Fig 5a). During the electrophysiological recording an additional stainless-steel wire placed on the neck muscle served as a reference and ground for the LAPS chip. The light delivered through the optical fiber was modulated at 200 Hz to generate alternating photocurrent in the LAPS. The magnitude of the photocurrent was determined by the channel conduction, which in turn was modulated by the external potentials. The slow-varying local field potentials (LFPs) were then recorded as the envelope functions of this photocurrent (Fig 5b). Spectral power analysis of the recorded traces revealed the presence of delta band oscillations, characteristic of the anesthetized brain [27] (Fig 5c).

In these initial experiments, a single optical source was used to address a single pixel within the LAPS chip. To fully realize mapping capability, this single optical source can raster scan the the backend of the multicore fiber, which can deliver light to designated spot and read out the localized information on the LAPS chip. Further more, in principle, the multicore fiber could also be coupled to a photonic chip with multiple outputs [28, 29] or an array of microscale light-emitting devices (μLEDs) [30], which would enable independent transmission of light through the individual waveguides and hence multi-spot recording of the field-modulated LAPS photocurrent.

## Conclusion

We have combined the field-effect sensor, i.e., the LAPS, with polymer-based multifuncitonal and multicore fibers as a miniature implantable device, to realize cost-effective and label-free electrophyiological recordings and mappings. In these devices, alternating optical signal delivered through one of the waveguides within the core of the polymer fiber illuminates the backside of the field-effect LAPS chip and produces an alternating photocurrent, the amplitude and phase of which are determined by the capacitance of the space charge region of the LAPS. The latter is modulated by the fluctuations in the local field potentials. This enables recording of electrophysiological signals as the amplitude envelopes of the photocurrents corresponding to the illuminated pixels of the LAPS chip. As this platform relies on the field effect rather than on optical signals from the labeled cells, it permits direct electrophysiological recording in deep-brain structures without the need for exogenously introduced fluorescent labels, and consequently it may be extended to model organisms that present a challenge to gene delivery techniques.

The spatial resolution of this platform is determined by the diameters of the waveguide pixels within the core of the multifunctional fiber and the ability to independently couple light into each of these pixels. Thermal drawing allows for reduction of polymer waveguide dimensions to few microns with conserved structures, as demonstrated in this study (waveguide diameter between 8 μm to 20 μm), and multiple drawing steps can be used to produce nanoscale features [22]. While introducing ferrules for optical coupling to each of the waveguides is impractical, deploying the raster scanning of a focused light beam into the each core of the light waveguide bundle serves as a solution. An alternative way may reside in integrated photonics approaches, akin to those routinely used in on-chip optical telecommunications [31], which may enable direct coupling to a multiplexed light source or an array of μLEDs.

Multifunctional fibers with similar composition have been previously applied for long-term electrophysiological studies in freely moving mice [24, 32]. In addition, LAPS chips are miniature and composed of biochemically inert materials [13, 15], and are expected to have negligible influence on the mechanical or chemical compatibility of such device with the surrounding tissue, suggesting their future utility in longitudinal studies in behaving subjects.

With configured light delivery system, this fiber-coupled field-effect platform may offer a neural recording approach complementary to the voltage readout with passive electrodes [3], optical imaging with gradient index endoscopes [4] and photometric readout of bulk fluorescence [33]. As the sensing surface of the LAPS chip can, in principle, be functionalized with reactive moieties such as enzymes [34], our device may be configured for detection of neurotransmitters and metabolites providing spatially resolved neurochemical profiles and offering insights into circuit function beyond local field potentials.

## Supporting information

S1 Fig Thermal drawing temperature, tension, stress and diameter of the PC/PMMA optical fiber. **a.** Adjustment of the drawing temperature during the process. **b.** Adjustment of the drawing tension during the thermal drawing. **c.** Stress experienced by the PC/PMMA fiber during the thermal drawing. **d.** Diameter of the fiber measured during the drawing process.
(TIF)

S2 Fig Thermal drawing temperature, tension, stress and diameter of the multifunctional fiber with optical waveguide bundle and BiSn electrodes. **a.** Adjustment of the drawing temperature during the process. **b.** Adjustment of the drawing tension during the thermal drawing. **c.** Stress experienced by the multifunctional fiber during the thermal drawing. **d.** Diameter of the fiber measured during the drawing process.
(TIF)

S3 Fig Cross-section of the fiber with a ring of multiple Sn electrodes and hollow channel, reprinted with permission from reference [23]. (TIF)
