# Supplementary figures and images for: Polymer-fiber-coupled field-effect sensors for label-free deep brain recordings
Source: PLoS One. 2020 Jan 24;15(1):e0228076. doi: 10.1371/journal.pone.0228076 (PMC6980412; doi:10.1371/journal.pone.0228076)

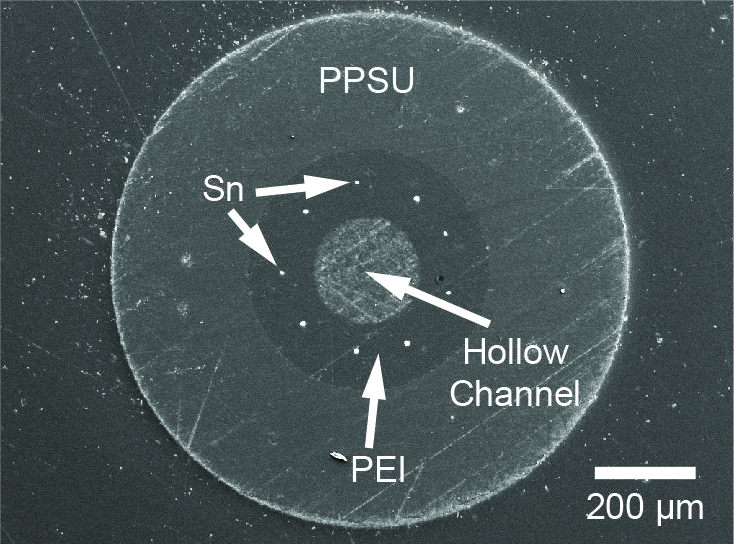

Supplement: S3 Fig — (TIF) [file pone.0228076.s003.tif]
